# Cross-Cultural Adaptation and Validation of the Mini-Eating and Drinking Ability Classification System for Korean Children with Cerebral Palsy Aged 18–36 Months

**DOI:** 10.3390/children12101348

**Published:** 2025-10-07

**Authors:** You Gyoung Yi, Seoyon Yang, Jeong-Yi Kwon, Dong-wook Rha, Juntaek Hong, Ja Young Choi, Eun Jae Ko, Bo Young Hong, Dae-Hyun Jang

**Affiliations:** 1 Department of Rehabilitation Medicine, Ewha Womans University Seoul Hospital, College of Medicine, Ewha Womans University, Seoul 07804, Republic of Korea; lyk861124@gmail.com (Y.G.Y.); seoyonyang@gmail.com (S.Y.); 2 Department of Physical and Rehabilitation Medicine, Samsung Medical Center, Sungkyunkwan University School of Medicine, Seoul 06351, Republic of Korea; 3 Department and Research Institute of Rehabilitation Medicine, Yonsei University College of Medicine, Seoul 03722, Republic of Koreaghdwnsxor@yuhs.ac (J.H.); 4 Department of Rehabilitation Medicine, Chungnam National University College of Medicine, Daejeon 34134, Republic of Korea; 5 Department of Rehabilitation Medicine, Asan Medical Center, College of Medicine, University of Ulsan, Seoul 05505, Republic of Korea; 6 Department of Rehabilitation Medicine, St. Vincent’s Hospital, College of Medicine, The Catholic University of Korea, Seoul 16247, Republic of Korea; 7 Department of Rehabilitation Medicine, Incheon St. Mary’s Hospital, College of Medicine, The Catholic University of Korea, Seoul 21431, Republic of Korea

**Keywords:** cerebral palsy, Mini-EDACS, eating and drinking ability, feeding difficulties, dysphagia, functional classification, reliability, validity

## Abstract

**Highlights:**

**What are the main findings?**

- The Korean version of the Mini-Eating and Drinking Ability Classification System (Mini-EDACS) demonstrated excellent inter-rater reliability between caregivers and clinicians.
- Construct validity was supported by significant correlations between Mini-EDACS levels and other functional classification systems (GMFCS, Mini-MACS, CFCS, VFCS, and FOIS-C).

**What is the implication of the main finding?**

- The Korean Mini-EDACS provides a reliable and valid framework to classify feeding and swallowing abilities in children with cerebral palsy aged 18–36 months.
- This tool enables standardized communication between clinicians and caregivers and may guide early identification of children needing feeding support.

**Abstract:**

Background/Objectives: Feeding and swallowing difficulties are common in young children with cerebral palsy (CP), yet no validated tool has been available in Korea for those under 3 years. The Mini-Eating and Drinking Ability Classification System (Mini-EDACS) was designed for children aged 18–36 months. This study aimed to translate the Mini-EDACS into Korean and evaluate its reliability and validity. Methods: Translation followed international guidelines, including forward–backward translation and Delphi consensus with experts in pediatric dysphagia. Forty-eight children with CP (mean age 27.1 ± 5.0 months) were assessed. Caregivers and speech–language pathologists (SLPs) independently rated Mini-EDACS and assistance levels. Inter-rater reliability was examined using Cohen’s κ. Construct validity was tested by Spearman’s correlations with the Gross Motor Function Classification System (GMFCS), Mini-MACS, the Communication Function Classification System (CFCS), the Visual Function Classification System (VFCS), and the Functional Oral Intake Scale for Children (FOIS-C). Results: Agreement between caregivers and SLPs was excellent (κ = 0.90; weighted κ = 0.98). Assistance-level ratings also showed almost perfect concordance (κ = 0.97). Mini-EDACS correlated strongly with FOIS-C (ρ = −0.86, *p* < 0.001) and with assistance levels (ρ = 0.81, *p* < 0.001). Moderate-to-strong positive correlations were observed with GMFCS (ρ = 0.56), Mini-MACS (ρ = 0.64), CFCS (ρ = 0.61), and VFCS (ρ = 0.61), supporting construct validity. Conclusions: The Korean Mini-EDACS is a reliable and valid tool for classifying eating and drinking abilities in children with CP under 3 years. It enables standardized communication between caregivers and clinicians, complements existing functional classification systems, and may facilitate earlier identification and intervention for feeding difficulties.

## 1. Introduction

Feeding and swallowing difficulties are common in children with cerebral palsy (CP) and can lead to malnutrition, growth impairment, respiratory complications, and reduced quality of life [1,2]. Previous research indicates that oropharyngeal dysphagia (OPD) often persists during early childhood, with gross motor function closely related to its severity and progression. For example, longitudinal studies have shown that more than half of preschool-aged children with CP exhibit OPD between 18 and 36 months of age, with severity remaining relatively stable over time, and gross motor function emerging as a strong predictor of both its presence and persistence [3].

The Eating and Drinking Ability Classification System (EDACS) was originally developed to provide a standardized functional framework for classifying eating and drinking performance in individuals with CP aged three years and older [4]. It categorizes abilities into five ordinal levels based on safety and efficiency and incorporates a three-level scale describing the level of assistance required [5,6]. EDACS has since been translated and validated in multiple languages, including Korean [7], Swedish [8], Greek [9], and Spanish [1], with studies consistently demonstrating excellent reliability and validity across diverse cultural contexts. These cross-cultural adaptations have facilitated its adoption in both clinical practice and research; however, studies have been conducted in populations aged ≥ 3 years (and, in some cases, adults), thereby leaving an unmet need for validated assessment tools applicable to younger children.

To address this gap, Sellers et al. developed the Mini-EDACS, a modified version of EDACS tailored for children aged 18–36 months [10]. This version retains the conceptual framework of EDACS but incorporates age-appropriate descriptors reflecting the developmental stage of toddlers. Validation work on the Mini-EDACS is still limited to two studies—the original development and validation in English [10] and a Dutch translation and validation study by van der Klift et al. [11]—both demonstrating good reliability, construct validity, and applicability.

In Korea, the EDACS for children aged ≥ 3 years was recently validated [7], but no Korean version of the Mini-EDACS exists, and its psychometric properties have not been examined in Korean toddlers with CP. Importantly, no validated instrument is currently available in Korea—or elsewhere in Asia—for the assessment of eating and drinking abilities in children younger than three years, underscoring the novelty and significance of this study. Given the Korean clinical emphasis on early identification and multidisciplinary management of feeding difficulties, a culturally adapted tool is essential to standardize assessments and support accurate communication between caregivers and clinicians.

The objective of this study was to translate and culturally adapt the Mini-EDACS into Korean and to examine its measurement properties in children with CP aged 18–36 months. We hypothesized that the Korean Mini-EDACS would demonstrate high inter-rater agreement between caregivers and professionals and exhibit significant correlations with established functional classification systems, supporting its validity as a clinical and research instrument.

## 2. Materials and Methods

### 2.1. Study Participants

Children aged 18–36 months with a confirmed diagnosis of CP were eligible for inclusion if they were accompanied by a parent or primary caregiver capable of understanding and completing Korean-language questionnaires. Exclusion criteria were children younger than 18 months or older than 36 months at the time of assessment, as well as refusal by the child’s caregiver or legal guardian to participate. The required sample size was determined based on the primary outcome of inter-rater reliability of the Korean Mini-EDACS. Using the method proposed by Walter [12], we assumed an expected ICC of 0.78, a minimum acceptable ICC of 0.60, two raters, a two-sided α of 0.05, and statistical power of 0.80. Under these assumptions, the minimum required sample was estimated at approximately n = 44. To further enhance the precision of reliability estimates and ensure robustness of construct validity analyses (correlation with other functional scales), we set the final target sample at 48 children with cerebral palsy. This sample size also provides at least 80% power to detect correlations of moderate magnitude (ρ = 0.40) at the 0.05 significance level. Therefore, recruiting 48 participants was considered sufficient and appropriate for the aims of this study.

### 2.2. Translation and Cultural Adaptation

Permission to translate and use the Mini-EDACS in Korean was obtained from the original developer (Diane Sellers, Sussex Community NHS Foundation Trust) prior to study initiation. The original English Mini-EDACS was translated into Korean following internationally recognized guidelines for cross-cultural adaptation of self-report measures [13]. First, two independent bilingual translators with clinical experience in pediatric rehabilitation each produced a forward translation. The two Korean drafts were then reconciled into a single version through consensus meetings, with discrepancies resolved by discussion and reference to the source instrument’s intent. Next, a separate bilingual translator—blinded to the original instrument—performed a backward translation into English to check for semantic drift.

Content validity of the Korean Mini-EDACS was evaluated using a Delphi survey with an expert panel comprising eight pediatric rehabilitation physicians (each with over 10 years of clinical experience in pediatric dysphagia rehabilitation) and two speech–language pathologists. Experts rated each item on a 7-point Likert scale (1 = strongly disagree to 7 = strongly agree) regarding clarity, cultural appropriateness, and conceptual equivalence. An open-ended question was also included to collect qualitative feedback and suggestions for improvement. Two or more Delphi rounds were conducted, and items were revised iteratively until at least 80% of experts rated each item as 6 or 7, in line with consensus-based methodological standards for evaluating content validity [14].

### 2.3. Data Collection and Outcome Measures

Participants were recruited through the Korean Cerebral Palsy Registry (KCPR) network between December 2024 and August 2025. Caregivers were informed of the study purpose and procedures, and written informed consent was obtained prior to participation. Baseline demographic and clinical information was obtained from the KCPR, including diagnosis, birth history, neuroimaging findings, and functional classification levels using the Gross Motor Function Classification System (GMFCS) [15], Mini-Manual Ability Classification System (Mini-MACS) [16], Communication Function Classification System (CFCS) [17], and Visual Function Classification System (VFCS) [18]. Feeding ability was assessed independently by two raters—a researcher and the child’s caregiver—using the Korean version of the Mini-Eating and Drinking Ability Classification System (Mini-EDACS), which categorizes eating and drinking performance into five ordinal levels based on safety and efficiency. All Mini-EDACS assessments were conducted by a single speech–language pathologist who was not the treating clinician for any of the children. Although the assessor was acquainted with some participants through the registry, they were not responsible for clinical care, thereby minimizing potential bias. CP subtypes were classified according to SCPE guidelines [19], based on the predominant neurological finding. Children showing both spastic and dyskinetic features without a clearly dominant presentation were categorized as unclassified. Caregivers received written instructions before completing their evaluations, and no further assistance was provided during the assessment. Additional assessments included the Functional Oral Intake Scale for Children (FOIS-C), a validated five-level scale evaluating functional oral intake [20]. The FOIS-C encompasses both the route of feeding and the degree of oral intake. Level 1 indicates nothing by mouth; Level 2 describes tube dependence with only minimal oral intake; Level 3 represents tube feeding combined with consistent oral intake contributing significantly to caloric needs; Level 4 denotes a total oral diet that still requires special preparation or compensations (e.g., grinding, thickening, or nutritional supplementation) or failure to progress beyond bottle feeding after 12 months of age; and Level 5 corresponds to a total oral diet without special preparation or compensations. This scale has been shown to be reliable and valid for assessing oral intake in children with cerebral palsy and other swallowing disorders [20]. The GMFCS, Mini-MACS, CFCS, and VFCS were used to classify gross motor, manual ability, communication, and visual function, respectively, for concurrent validity analyses.

### 2.4. Statistical Analysis

Inter-rater reliability between caregiver and researcher ratings on the Mini-EDACS was evaluated using weighted kappa coefficients with both linear and quadratic weighting schemes. Kappa values between 0.61 and 0.80 were interpreted as indicating substantial agreement, and values greater than 0.80 as indicating almost perfect agreement. Construct validity was assessed by examining Spearman’s rank correlations between Mini-EDACS scores and those from the FOIS-C, GMFCS, Mini-MACS, CFCS, and VFCS. A correlation coefficient of 0.50 or higher was considered indicative of good validity. All statistical analyses were two-tailed with a significance level of α = 0.05, and were performed using SPSS (Version 31, IBM Corp., Armonk, NY, USA) or R software, version 4.4.1 (R Foundation for Statistical Computing, Vienna, Austria).

## 3. Results

### 3.1. Translation and Cultural Adaptation

Of the eleven items evaluated, four achieved consensus in the first round, while seven required revision based on expert comments. These revisions mainly involved refining age-appropriate descriptors, clarifying level boundaries, and adapting terminology to the Korean clinical and cultural context (e.g., consistent translation of “tiring,” clarification of “selectively controlling mouth opening and tongue movement,” and addition of illustrations for cup types). After two Delphi rounds, consensus was reached for all items except one, which required a third round. Ultimately, all 11 items achieved ≥ 80% agreement, confirming the content validity of the Korean Mini-EDACS. In the final round, all items reached the predefined consensus threshold (Supplementary Table S1), with ≥80% of experts rating them as 6 or 7, thereby confirming the content validity of the Korean Mini-EDACS.

### 3.2. Participant Characteristics

Children with CP aged 18–36 months were recruited through the KCPR network. A total of 48 families were approached, and all provided informed consent to participate, with the children having a mean age of 27.1 ± 5.0 months (Table 1). The participants comprised 20 boys (41.7%) and 28 girls (58.3%). Regarding gross motor function, 12 children (25.0%) were classified as GMFCS level I, 14 (29.2%) as level II, 10 (20.8%) as level III, and 6 (12.5%) each as levels IV and V. Manual ability, as assessed by the Mini-MACS, showed a wider distribution: 2 children (4.2%) at level I, 11 (22.9%) at level II, 18 (37.5%) at level III, 8 (16.7%) at level IV, and 9 (18.8%) at level V. CFCS was assessed only in children aged ≥24 months. Among these, 4 children (8.3%) were classified as level I, 5 (10.4%) as level II, 17 (35.4%) as level III, 7 (14.6%) as level IV, and 4 (8.3%) as level V. Feeding performance, measured by the FOIS-C, showed that 2 children (4.2%) were at level I, 1 (2.1%) at level II, 1 (2.1%) at level III, 18 (37.5%) at level IV, and 26 (54.2%) at level V. Feeding ability using the Korean Mini-EDACS showed that 28 children (58.3%) were classified at Level I, 4 (8.3%) at Level II, 7 (14.6%) at Level III, 4 (8.3%) at Level IV, and 5 (10.4%) at Level V. Overall, the cohort represented a heterogeneous group in terms of motor, manual, communication, and feeding abilities, reflecting the clinical diversity of young children with CP.

### 3.3. Inter-Rater Reliability

Agreement between caregiver and SLP ratings of the Korean Mini-EDACS was excellent (Table 2). The unweighted Cohen’s κ was 0.90, indicating almost perfect agreement. When the ordinal structure of the scale was taken into account, the weighted κ values were even higher—0.95 with linear weighting and 0.98 with quadratic weighting—demonstrating that disagreements, when present, were minor and usually limited to adjacent levels. These findings support the robustness of the Korean Mini-EDACS as a reliable measure for assessing eating and drinking abilities in young children with CP. As shown in Table 3, caregiver and SLP ratings of assistance levels demonstrated almost perfect agreement, with only one discrepancy observed between the Independent and Requires assistance categories.

### 3.4. Construct Validity

Correlation analyses demonstrated significant associations between the Korean Mini-EDACS and other functional classification systems (Table 4). Mini-EDACS levels were strongly and negatively correlated with the Functional Oral Intake Scale for Children (FOIS-C; ρ = −0.86, 95% CI −0.95 to −0.73, *p* < 0.001), indicating that children with better oral intake abilities tended to be classified at lower (better)Mini-EDACS levels.

Mini-EDACS levels also showed moderate-to-strong positive correlations with motor, manual, communication, and visual function classifications. Specifically, correlations were observed with the GMFCS (ρ = 0.56, 95% CI 0.30–0.77, *p* < 0.001), Mini-MACS (ρ = 0.64, 95% CI 0.43–0.80, *p* < 0.001), CFCS (ρ = 0.61, 95% CI 0.38–0.80, *p* < 0.001), and VFCS (ρ = 0.61, 95% CI 0.37–0.77, *p* < 0.001). The strongest positive correlation was found between the Mini-EDACS and its corresponding assistance level ratings (ρ = 0.81, 95% CI 0.71–0.87, *p* < 0.001), suggesting consistent interpretation between the two scales.

## 4. Discussion

In the present study, 44 of 48 participants presented with spastic CP, while only four children had dyskinetic or unclassifiable types. This distribution reflects the clinical epidemiology in Korea, where the spastic subtype constitutes the majority of CP cases [21] and where survival of preterm infants has contributed to changing prevalence patterns [22].

The participants in this study represented a broad spectrum of functional abilities, reflecting the heterogeneity of young children with CP. Although their mean age was around 27 months, their gross motor function ranged from GMFCS I to V, and manual abilities spanned all five Mini-MACS levels. More than half of the children were classified as GMFCS I–II, reflecting relatively mild mobility impairment, yet their feeding abilities varied widely. Notably, a majority (about 92%) did not require tube feeding (FOIS-C levels IV–V), indicating that most in the present study had relatively preserved feeding function despite their neurological impairment. Compared with the Mini-EDACS development study [10], which included predominantly more severely affected children (84% GMFCS IV–V, with 7% requiring tube feeding, all GMFCS V), our cohort represented relatively higher functioning children, with over half at GMFCS I–II. This contrast highlights how the Mini-EDACS can be applied across diverse CP populations, from severely impaired, tube-fed toddlers to higher functioning cohorts, and underscores the value of integrating feeding-specific classification alongside other functional systems to capture the full clinical heterogeneity of early childhood CP.

Notably, the communication function showed a wide distribution, with substantial proportions of children classified across CFCS levels. Visual function in our cohort was largely preserved (VFCS I in 42/48, 87.5%), with only a small subset showing impairment (VFCS II in 3/48, 6.3%; VFCS IV in 3/48, 6.3%). None of the children rated VFCS I were classified as Mini-EDACS Level V, whereas all Mini-EDACS Level V cases occurred among those with VFCS II or IV (Supplementary Table S2). This pattern is consistent with the construct that visual function can facilitate safe, efficient feeding (e.g., orienting, utensil use, and timing of swallows), and it aligns with the moderate correlation observed between Mini-EDACS and VFCS in our sample. At the same time, the marked ceiling in VFCS (predominance of level I) may limit granularity and could attenuate estimates of association; future studies enriched for children with visual impairment would help clarify dose–response relationships across the full VFCS spectrum. In contrast, manual dexterity was more broadly affected, with over half classified as Mini-MACS level III or worse—consistent with the well-recognized vulnerability of fine motor control, even in mildly affected CP. Taken together, this functional profile underscores the importance of assessing multiple domains in parallel. The Mini-EDACS provides a feeding-specific complement to established systems, thereby offering a more comprehensive view of each child. The heterogeneity observed across these domains in our cohort highlights the necessity of employing such classification systems to fully capture the clinical diversity of early childhood CP.

### 4.1. Inter-Rater Reliability

Our findings show that the Korean Mini-EDACS can be applied with excellent inter-rater reliability between caregivers and professionals. The unweighted Cohen’s κ was 0.90, reflecting almost perfect agreement beyond chance. When the ordinal nature of the Mini-EDACS categories was taken into account, the weighted κ values increased further, reaching up to 0.98. This indicates that caregiver and SLP ratings were virtually identical for the majority of participants, with the few observed discrepancies limited to minor differences between adjacent levels. This level of agreement is higher than that reported in the original English EDACS development study, where parental ratings tended to only moderately agree with therapists (κ = 0.45) [10]. In this study, parents generally either agreed with clinicians or, when discrepancies occurred, tended to rate their child as slightly more capable—typically assigning one level higher—than the clinician’s rating [10]. A similar pattern was observed in our data—in the few cases of discrepancy, caregivers rated the child at a better level than the SLP—but such cases were very rare, indicating a stronger consensus than previous studies. Our substantially higher κ suggests that the translation and adaptation of Mini-EDACS into Korean was successful in producing clear, parent-friendly descriptors and that, with appropriate guidance or training, caregivers can reliably use this tool. It is encouraging that other validation studies have also achieved high inter-rater reliability after cultural adaptation of EDACS; for example, a European study reported κ = 0.82 between professionals and parents, with nearly all disagreements within one level [6].

Furthermore, the agreement on the Mini-EDACS assistance levels was virtually perfect in our study (only 1 out of 48 ratings differed between caregiver and SLP). This means both raters nearly always agreed on whether a child was independent, required assistance, or was totally dependent for eating/drinking. Such consistency highlights the clarity of the assistance level definitions and their ease of use for non-professionals. Prior research has similarly found very high reliability for the EDACS assistance scale (κ = 0.89) alongside the main EDACS levels [6]. Taken together, these data demonstrate the robustness of the Korean Mini-EDACS: not only can trained clinicians apply it reliably, but caregivers—who are intimately familiar with their child’s feeding—can also classify their child’s abilities with a high degree of agreement. The high level of agreement between caregivers and professionals suggests that the Mini-EDACS could be particularly valuable in clinical settings with limited professional observation time, as it enables the incorporation of caregiver reports while maintaining the accuracy of assessment.

### 4.2. Construct Validity

The Mini-EDACS levels demonstrated a consistent profile of correlations with other measures, supporting its construct validity. Most notably, there was a strong inverse correlation with the FOIS-C (Spearman ρ = −0.86, *p* < 0.001). This high negative correlation indicates that children classified at better Mini-EDACS levels (lower numerical levels, indicating greater safety and efficiency in eating and drinking) tended to have higher FOIS-C levels (indicating more advanced oral intake). In other words, the Mini-EDACS levels in our study closely reflected children’s actual feeding status. Those classified at lower levels—indicating safer and more efficient eating and drinking—were consistently on full oral diets, whereas children at higher Mini-EDACS levels showed more restricted oral intake or required alternative feeding routes. This pattern is consistent with findings from prior research on the original EDACS. For instance, Hyun et al. [23] reported that EDACS levels in adults were significantly correlated with FOIS scores and swallowing-related quality of life. Likewise, a recent study in the Netherlands translated and validated the Mini-EDACS in preschool-aged children with CP, reporting almost perfect inter-rater reliability between clinicians (weighted κ = 0.83) and substantial agreement between clinicians and parents (κ = 0.70–0.77). Construct validity was also supported by significant correlations with the Pediatric Eating Assessment Tool and the Montreal Children’s Hospital Feeding Scale [11]. Our findings corroborate and extend these results by providing the first cross-cultural validation of the Mini-EDACS in Korean.

Mini-EDACS levels also demonstrated a strong association with the corresponding assistance ratings (ρ = 0.81). Children classified at higher Mini-EDACS levels almost invariably required greater support during meals, reflecting the conceptual premise that feeding safety and efficiency decline as dependence increases. Similar findings have been reported for the original EDACS, where higher levels were associated with partial or full caregiver assistance [10].

In addition to feeding-specific measures, we examined correlations with broader functional classification systems. We found moderate, statistically significant positive correlations between Mini-EDACS level and the severity levels of gross motor function (GMFCS, ρ = 0.56), manual ability (Mini-MACS, ρ = 0.64), communication (CFCS, ρ = 0.61), and visual function (VFCS, ρ = 0.61) (all *p* < 0.001). These correlations indicate that children with more severe eating/drinking impairments were more likely to have higher levels of motor, hand, communication, and visual impairments. This observation is biologically consistent with the pathophysiology of CP, in which greater severity frequently reflects more extensive neurological deficits; accordingly, dysphagia severity tends to co-vary with both motor limitations and cognitive impairment [24]. Our findings are in line with previous observations that feeding difficulties often co-occur with other functional limitations. For instance, one study reported a strong relationship between GMFCS level and EDACS level in children with CP [23], and others have noted that children in the most severe GMFCS categories frequently present with significant oropharyngeal dysphagia and malnutrition risks [25,26]. Some children with relatively mild ambulatory impairment may nonetheless exhibit profound oral–motor or swallowing difficulties—for example, individuals with athetoid CP who are able to walk independently yet struggle with coordinated swallowing. Conversely, children with severe limitations in mobility may still manage oral feeding if bulbar functions are relatively preserved. These findings underscore that eating and drinking ability constitute a distinct functional domain influenced by oromotor control and sensory integration, rather than merely reflecting gross motor capacity. Therefore, the Mini-EDACS adds valuable, complementary insights by identifying feeding difficulties unaccounted for by other classification systems.

### 4.3. Study Limitations

This study has several limitations. First, the sample size was modest (48 children) and unevenly distributed across severity levels; only five children were classified as Mini-EDACS Level V. As a result, the most severely impaired children—those who are non-ambulatory or non-oral feeders—were underrepresented, limiting the generalizability of our findings to the broader CP population. Larger studies with a more severity-diverse cohort are warranted. Second, caregiver–therapist agreement may have been enhanced by study conditions. Caregivers received clear explanations of Mini-EDACS definitions, which could reduce variability compared to routine practice. This highlights the importance of caregiver training and the possible benefit of illustrative examples when applying the classification at home. Third, validation was limited to cross-sectional correlations with other functional classifications. Although these analyses support construct validity, predictive validity, and clinical outcomes—such as aspiration events, growth, or respiratory complications—were not examined. Nor did we include an objective reference standard such as a videofluoroscopic swallowing study, as no single gold standard exists for functional feeding in this age group. Another limitation is that the study employed a cross-sectional design, and short-term test–retest reliability of the Korean Mini-EDACS was not assessed. Future studies should examine whether Mini-EDACS ratings remain stable across repeated caregiver assessments over time. Additionally, most participants had spastic CP, with only a few presenting with dyskinetic or unclassifiable types. This distribution reflects the Korean epidemiology of CP, where spastic CP predominates due to improved preterm survival [21,22]. Nevertheless, the limited representation of non-spastic types constrains generalizability, and further studies with more diverse samples are needed to validate the Mini-EDACS across all CP phenotypes. Finally, professional ratings were conducted by a single speech–language pathologist, which precluded assessment of inter-professional reliability. Future studies should include multiple professional raters to determine whether agreement is maintained across different clinicians.

## 5. Conclusions

This study demonstrated that the Korean version of the Mini-EDACS is a reliable and valid instrument for classifying eating and drinking abilities in children with cerebral palsy aged 18–36 months. To our knowledge, this is the first validated tool for this age group in Korea and, more broadly, in Asia. By providing a culturally adapted classification system, the Korean Mini-EDACS enables clinicians and caregivers to identify feeding difficulties earlier, thereby supporting timely interventions that may reduce risks such as malnutrition, growth impairment, and respiratory complications. Beyond its immediate clinical utility, this tool expands the functional classification framework available for young children with CP, complementing established measures of gross motor, manual, and communication function. Future longitudinal research is warranted to determine whether Mini-EDACS levels in early childhood can predict longer-term outcomes, including nutritional trajectories, growth, and respiratory health.

## Figures and Tables

**Table 1:** Demographic and Clinical Characteristics of Participants with Cerebral Palsy.

Variable	n (%)/Mean ± SD
Age, months	27.1 ± 5.0
Sex	Male: 20 (41.7%), Female: 28 (58.3%)
GMFCS level	
I	12 (25.0)
II	14 (29.2)
III	10 (20.8)
IV	6 (12.5)
V	6 (12.5)
Mini-MACS level	
I	2 (4.2)
II	11 (22.9)
III	18 (37.5)
IV	8 (16.7)
V	9 (18.8)
CFCS level ^1^	
I	4 (10.8)
II	5 (13.5)
III	17 (45.9)
IV	7 (18.9)
V	4 (10.8)
FOIS-C level	
I	2 (4.2)
II	1 (2.1)
III	1 (2.1)
IV	18 (37.5)
V	26 (54.2)
Mini-EDACS level	
I	28 (58.3)
II	4 (8.3)
III	7 (14.6)
IV	4 (8.3)
V	5 (10.4)
Predominant motor type	
Spastic bilateral	37 (77.1)
Spastic unilateral	7 (14.6)
Dyskinetic	2 (4.2)
Unclassifiable	2 (4.2)

^1^ CFCS: evaluated only in children aged ≥24 months.

**Table 2:** Agreement between caregiver and SLP ratings for mini-EDACS levels.

Caregiver Rating	SLP Rating					
	Level I	Level II	Level III	Level IV	Level V	Total
Level I	28	0	0	0	0	28
Level II	0	4	0	0	0	4
Level III	0	0	7	1	0	8
Level IV	0	0	0	3	2	5
Level V	0	0	0	0	3	3
Total	28	4	7	4	5	48

**Table 3:** Agreement between caregiver and SLP ratings for mini-EDACS assistance levels.

Caregiver Rating	SLP Rating			
	Independent	Requires Assistance	Totally Dependent	Total
Independent	15	1	0	16
Requires assistance	0	17	0	17
Totally dependent	0	0	15	15
Total	15	18	15	48

**Table 4:** Spearman’s correlation coefficients between Korean Mini-EDACS and other functional classification systems.

Variable	ρ (Spearman’s)	95% CI for ρ	*p*-Value
FOIS-C	−0.86	−0.95–−0.73	<0.001
GMFCS	0.56	0.30–0.77	<0.001
Mini-MACS	0.64	0.43–0.80	<0.001
CFCS	0.61	0.38–0.80	<0.001
VFCS	0.61	0.37–0.77	<0.001

## Data Availability

The data presented in this study are available on request from the corresponding author. The data are not publicly available due to privacy and ethical restrictions.

## Supplementary Materials

The following supporting information can be downloaded at: https://www.mdpi.com/article/10.3390/children12101348/s1, Table S1: Delphi Survey Results for the Content Validity of the Korean Mini-EDACS; Table S2: Distribution of Mini-EDACS Levels Across Functional Classification Systems (GMFCS, Mini-MACS, CFCS, VFCS).

## Abbreviations

The following abbreviations are used in this manuscript:

CP	Cerebral palsy
EDACS	Eating and Drinking Ability Classification System
Mini-EDACS	Mini-Eating and Drinking Ability Classification System
FOIS-C	Functional Oral Intake Scale for Children
GMFCS	Gross Motor Function Classification System
Mini-MACS	Mini-Manual Ability Classification System
CFCS	Communication Function Classification System
VFCS	Visual Function Classification System
SLP	Speech–Language Pathologist
