# Supplementary material for: Cross-Cultural Adaptation and Validation of the Mini-Eating and Drinking Ability Classification System for Korean Children with Cerebral Palsy Aged 18–36 Months
Source: Children (Basel). 2025 Oct 7;12(10):1348. doi: 10.3390/children12101348 (PMC12564789; doi:10.3390/children12101348)
Supplement: Supplementary file 1 [file children-12-01348-s001.zip › children-3888582-supplementary.pdf]

**Supplementary table S1**. Delphi Survey Results for the Content Validity of the Korean Mini-EDACS

| Item | Round | Mean (±SD) | % ≥6 | Revision made | Final status |
|---|---|---|---|---|---|
| 1. Purpose clarity | R1 | 5.6 ± 0.9 | 60% | Age-appropriate descriptors refined | Accepted (R2: 6.7 ± 0.5, 100%) |
| 2. Background appropriateness | R1 | 5.8 ± 1.0 | 60% | Simplified background explanation | Accepted (R2: 6.8 ± 0.4, 100%) |
| 3. Key features | R1 | 5.7 ± 0.8 | 70% | Clarified major characteristics | Accepted (R2: 6.9 ± 0.3, 100%) |
| 4. User guidance clarity | R1 | 5.5 ± 1.1 | 50% | Instruction wording revised | Accepted (R2: 6.6 ± 0.5, 100%) |
| 5. Level I–V descriptors | R1 | 5.9 ± 0.7 | 70% | Added explanatory notes & examples | Accepted (R2: 6.8 ± 0.4, 100%) |
| 6. Cultural/clinical appropriateness | R1 | 5.4 ± 1.2 | 50% | Terminology revised; illustrations (spout/valve cups) added | R2: 6.3 ± 0.6 (70%); R3: 6.8 ± 0.4 (100%) |
| 7. Level differentiation | R1 | 5.8 ± 0.9 | 60% | Boundary definitions improved | Accepted (R2: 6.9 ± 0.3, 100%) |
| 8. Considerations for 18–36 months | R1 | 6.3 ± 0.5 | 90% | None | Accepted |
| 9. Conceptual equivalence | R1 | 6.5 ± 0.6 | 90% | None | Accepted |
| 10. Item clarity overall | R1 | 6.6 ± 0.5 | 100% | None | Accepted |
| 11. Terminology consistency | R1 | 6.2 ± 0.7 | 85% | None | Accepted |

**Supplementary Table S2**. Distribution of Mini-EDACS Levels Across Functional Classification Systems (GMFCS, Mini-MACS, CFCS, VFCS)

| | | Mini-EDACS | | | | | |
|---|---|---|---|---|---|---|---|
| **Classification System** | | **I** | **II** | **III** | **IV** | **V** | **Total** |
| **GMFCS** | I | 11 | 0 | 0 | 1 | 0 | 12 |
| | II | 8 | 2 | 3 | 0 | 1 | 14 |
| | III | 8 | 1 | 1 | 0 | 0 | 10 |
| | IV | 1 | 1 | 2 | 2 | 0 | 6 |
| | V | 0 | 0 | 1 | 1 | 4 | 6 |
| **Mini-MACS** | I | 2 | 0 | 0 | 0 | 0 | 2 |
| | II | 10 | 1 | 0 | 0 | 0 | 11 |
| | III | 12 | 2 | 2 | 1 | 1 | 18 |
| | IV | 3 | 1 | 4 | 0 | 0 | 8 |
| | V | 1 | 0 | 1 | 3 | 4 | 9 |
| **CFCS** | I | 4 | 0 | 0 | 0 | 0 | 4 |
| (≥24 mo only) | II | 5 | 0 | 0 | 0 | 0 | 5 |
| | III | 12 | 2 | 1 | 1 | 1 | 17 |
| | IV | 2 | 2 | 2 | 1 | 0 | 7 |
| | V | 0 | 0 | 1 | 1 | 2 | 4 |
| **VFCS** | I | 28 | 4 | 6 | 4 | 0 | 42 |
| | II | 0 | 0 | 1 | 0 | 2 | 3 |
| | IV | 0 | 0 | 0 | 0 | 3 | 3 |
